# Association of Frailty Status with Semi-Targeted Metabolomics and Untargeted Proteomics in Older Adults Referred for Physical Therapy in a Geriatric Clinic

**DOI:** 10.3390/nu18203336

**Published:** 2026-10-12

**Authors:** Dakota J. Batchek, David H. Lynch, Laura Herring, Whitney L. Stutts, Scott P. Lyons, Crystal L. Pace, Danae C. Gross, Hillary B. Spangler, John A. Batsis

**Affiliations:** 1 Department of Nutrition, Gillings School of Global Public Health, University of North Carolina at Chapel Hill, Chapel Hill, NC 27599, USA; dakota_batchek@med.unc.edu (D.J.B.); dcgross@unc.edu (D.C.G.); 2 Division of Geriatric Medicine, School of Medicine, University of North Carolina at Chapel Hill, Chapel Hill, NC 27514, USA; david_lynch@med.unc.edu (D.H.L.); hillary.spangler@unchealth.unc.edu (H.B.S.); 3 Metabolomics and Proteomics Core, Department of Pharmacology, University of North Carolina at Chapel Hill, Chapel Hill, NC 27599, USA; laura_herring@med.unc.edu (L.H.); wlstutts@email.unc.edu (W.L.S.); scott_lyons@med.unc.edu (S.P.L.); clpace@email.unc.edu (C.L.P.)

**Keywords:** frailty, metabolomics, proteomics, older adults, physical function, biomarkers

## Abstract

**Background**: Older adults are vulnerable to frailty, but the biological mechanisms are unclear. Metabolomics and proteomics may help characterize the biological basis of frailty that can be used to target future interventions. **Methods**: This study is a cross-sectional analysis of baseline anthropometric, metabolomic, and proteomic data collected from older adults enrolled in a 12-week prospective physical therapy intervention. Frailty was classified using a modified Cumulative Deficit Index (CDI; 37 variables) and the Fried Frailty Index (5 variables). Gait speed, grip strength, and patient-reported outcome surveys were administered. Baseline plasma was analyzed via semi-targeted metabolomics (capillary electrophoresis-mass spectrometry) and untargeted proteomics (LC-MS/MS) using the CDI-defined frailty classification. **Results**: Mean age was 77.1 ± 5.5 (33% female). Using the CDI, 12 participants had pre-frailty and 6 had frailty. Participants with frailty had worse gait speed (*p* = 0.01), instrumental activity of daily living impairments (*p* = 0.005), and lower physical activity (*p* = 0.006). Metabolomics showed nicotinamide as decreased (*p* = 0.0066; log_2_FC = −1.19; FDR correction q = 0.753). Amino acid metabolites showed strong, positive correlations with weight, but negative with gait speed. Proteomics showed 65 differentially expressed proteins by frailty status (FDR-adjusted q-value < 0.05), 53 of which also with a |log_2_FC| ≥ 0.6 (26 higher in participants with frailty, and 27 higher in participants with pre-frailty). Specifically, histone proteins were elevated in participants with frailty. In exploratory, descriptive correlation analyses, patterns of association between protein levels and physical function measures differed between pre-frail and frail status, including apparent shifts in correlation direction and magnitude, most notably for gait speed and dietary scores. **Conclusions**: Histone proteins and nicotinamide may warrant further investigation as candidate exploratory markers in relation to frailty. However, our cross-sectional pilot study is unable to accurately delineate a definitive mechanistic association. Moreover, future studies should investigate these biomarkers’ roles in frailty progression. Our findings suggest a potential signal, yet in light of the small sample size, should not be interpreted as causal in nature.

## 1. Introduction

Physical frailty is a geriatric syndrome characterized by diminished physiologic reserve and increased vulnerability stressors [1,2]. It affects approximately 4–15% of adults aged ≥65 years, with prevalence rates rising substantially with age [3,4,5]. Frailty is most commonly categorized using the Cumulative Deficit Index (CDI) or the Fried Frailty Index [6,7,8,9]. Regardless of the definition used, frailty is associated with impaired functional independence, increased risk of hospitalization, institutionalization, dementia, mortality, and reduced quality of life [6,9,10].

Understanding the biological mechanisms underlying frailty has emerged as a priority within the geroscience framework, which posits that targeting the hallmarks of aging may mitigate multiple age-related conditions simultaneously [11,12]. While prior studies have associated frailty to elevated inflammatory markers such as C-reactive protein (CRP), Interleukin-6 (IL-6), Tumor Necrosis Factor Alpha (TNF-α), and alterations in anabolic pathways including insulin-like growth factor-1 (IGF-1), these investigations have largely focused on individual biomarkers, and not the cumulative biomarker profiles analyzed in semi- and untargeted approaches [13]. Moreover, such approaches may not fully capture the complex and heterogeneous biological underpinnings of frailty that may help in characterizing specific phenotypes of reduced physical function [14,15,16].

Multi-omics strategies, including metabolomics and proteomics, provide an emerging opportunity to characterize molecular structures associated with frailty and impaired physical function. These approaches may help characterize biological heterogeneity among older adults and identify candidate biomarkers or entire biomarker profiles for risk stratification and intervention targeting [17,18,19,20]. However, measuring clinical frailty alongside multi-omics profiles remains limited, preventing the ability to understand ‘biological aging’ and ‘inflammaging.’ [21,22]. To address this gap, we analyzed samples from a single-arm pilot study of community dwelling adults aged ≥65 years referred to outpatient physical therapy. We conducted metabolomic and proteomic analyses on baseline samples (prior to physical therapy) and evaluated their association with frailty status and physical function. Given the limited understanding of how specific metabolites and proteins relate to frailty in older adults, this exploratory, hypothesis-generating study aimed to identify candidate metabolomic and proteomic analytes that differ across frailty status (defined by the CDI) and correlate with physical function measures [23,24,25,26]. A priori, our findings are intended to be interpreted as hypothesis-generating rather than evidence of independent association such as could be derived from a larger, fully powered study [23,24,25,26].

## 2. Materials and Methods

### 2.1. Study Setting and Participants

This is a cross-sectional analysis of baseline blood markers and physical function data collected as part of a 12-week prospective pilot study evaluating a physical therapy intervention, conducted at the University of North Carolina at Chapel Hill (UNC) Geriatric Specialty Clinic between June 2022 and March 2023. Although the data was analyzed from a multi-day study, all the data we analyzed was from baseline only and thus explicitly cross-sectional in nature. All participants provided written consent in accordance with the University of North Carolina at Chapel Hill (UNC) Institutional Review Board (IRB), study #22-1230. Consent was completed at the clinic or at an agreed-upon location.

Participants were eligible if they were ≥65 years of age, currently being cared for at the Geriatrics clinic, had at least two chronic medical conditions, and were referred by their clinical provider to a 12-week community- or university-based physical therapy (PT) program. Key exclusion criteria included life-threatening illnesses, or untreated psychiatric conditions that could interfere with participation, dementia, participation in another PT program, scheduled surgery in the next six months, or any of the following comorbidities due to risk of being unable to participate in the PT intervention: hospitalization for heart failure in past 6 months, history of advanced non-skin cancer (Stage III or IV), advanced liver failure, or chronic renal insufficiency on hemodialysis. No formal sample size or power calculation was performed prior to this study, given its pilot and exploratory nature. The sample size (n = 18) was determined by the number of eligible participants who provided informed consent and completed baseline blood draws and functional measures.

### 2.2. Study Measures

Baseline demographic characteristics, anthropometrics, exhaustion questions, and physical function were collected upon entry into the study. Weight was measured using a standardized digital scale [27]. Grip strength was assessed using a hand-held dynamometer with participants seated, elbow supported on a table, and palm facing up [28]. Two trials were performed in the dominant hand; the highest value (kg) was recorded [28]. Gait speed was assessed over a 4-m course at usual pace [29,30]. Subjective measures were completed in REDCap, an online data capture reporting system [31,32]. Instruments included were: Katz Activities of Daily Living (ADL), Lawton Instrumental Activities of Daily Living (IADL), Brief Resilience Scale (BRS), Community Healthy Activities Model Program for Seniors Physical Activity Scale (CHAMPS), Rapid Eating Assessment (REAP-S), Patient Activation Measure 13-item (PAM-13), and Pittsburgh Fatiguability Scale (PFS).

### 2.3. Blood Collection and Processing

Blood specimens were collected at baseline into ethylenediaminetetraacetic acid (EDTA) tubes. Samples were centrifuged at 4 °C for 15 min at 1500× *g* within 60 min of collection. One buffy coat and four plasma aliquots were stored at −80 °C until analysis.

### 2.4. Frailty Stratification

Participants were stratified at baseline using the CDI and Fried Frailty Index separately, and categorized as robust, pre-frail, or frail [6,8]. For the CDI, 37 binary health deficits were constructed (1 = deficit present; 0 = absent); proportions of deficits were then made by dividing the total number of deficits present by 37, all of which were consistent with previous guidance in creating a composite frailty measure [8]. Frailty categories were classified as: robust: <0.08; pre-frail: 0.08–0.24; and frail: ≥0.25 [8]. For the Fried Frailty Index, we used a modified Fried Frailty Phenotype with modified criteria and their supporting rationale, as described in the Appendix A. Specifically, we assessed exhaustion, gait speed, grip strength, weight loss, and physical activity (binary yes/no) [6]. Participants were classified as robust (no criteria), pre-frail (1–2 criteria), or frail (≥3 criteria) [6]. All metabolomic and proteomic data were analyzed using the CDI, rather than the Fried Index, for two reasons. First, as a deficit-accumulation measure spanning multiple health domains, the CDI is designed to capture cumulative physiological burden rather than a specific physical function phenotype, which we reasoned may be more broadly representative in terms of using with our molecular measures. Second, from a practical standpoint, the CDI classified participants into two groups (pre-frail and frail) in our cohort, while the Fried Index yielded three groups (robust, pre-frail, and frail), which combined with our small sample size would have left insufficient data per group for the omics comparisons. The decision to use the CDI as our primary frailty classification was not formally pre-specified. Instead, it was made prior to conducting the omics comparisons, once it became clear that the Fried Index’s three-category classification would leave insufficient data per group for these analyses—in addition to the conceptual rationale surrounding the CDI described above.

### 2.5. Anthropometric Statistical Analysis

All analyses of anthropometric data were conducted in R version 4.5.1 (2025-06-13 ucrt) and Microsoft Excel. Continuous variables are presented as mean ± SD or median (IQR), as appropriate, and were compared between groups using unpaired *t*-tests. Non-parametric equivalents were used where appropriate. Categorical variables are presented as n (%) and were compared between groups using Fisher’s exact test, given the small sample size and cell counts. A two-sided *p*-value of 0.05 was considered statistically significant. Given the pilot nature of this study, Cohen’s d effect sizes were calculated and interpreted as small (0.2), medium (0.5), or large (0.8) using both Microsoft Excel and R Studio software (v2026.09.0) [33]. Using methods proposed by Hedges and Olkin (1985), 95% confidence intervals for Cohen’s d effect sizes for smaller sample sizes were calculated to convey the precision of these estimates given sample size limitations [34]. Given the small sample size in this study, there were no formal assessments of normality and homogeneity of variance due to their limited power at low sample sizes such as these. Welch’s unpaired *t*-tests (unequal variances) were used for all continuous variable comparisons. Baseline demographic comparisons (Table 1 and Table A1) were presented as descriptive characteristics of our sample and not intended to address any hypotheses. Comparison data presented for each frailty index (Table 2 and Table A2 for CDI and Fried, respectively) were not corrected for multiple comparisons using Benjamin–Hochberg FDR correction, and thus should be interpreted as exploratory, hypothesis-generating signals as opposed to established associations.

### 2.6. Metabolomics Sample Preparation

Equal amounts of each plasma sample were combined to create a study-pooled quality control (SPQC) sample. Metabolites were extracted and filtered from human plasma with the ZipChip ACE RM Analysis Kit (908 Devices, Boston, MA, USA). Briefly, 20 µL of human plasma, processing blanks, calibration standards, and QC samples were plated onto a 96-well glass-fiber filter plate. Samples and QCs were extracted with 140 µL of RM Extraction Reagent (100% methanol containing heavy-labeled internal standards). The sample plate was sealed with foil and thoroughly mixed at 500 RPM (Thermo Mixer, Eppendorf, Hamburg, Germany) for 15 min. Samples were diluted with 40 µL of RM Focusing Reagent (ammonium acetate buffer), covered and thoroughly mixed at 500 RPM (Thermo Mixer) for 5 min. Extracted metabolites were filtered into a deep-well plate by centrifugation at 1000 RPM for CE-MS analysis.

### 2.7. Metabolomics CE-MS Analysis

Metabolites were separated by capillary electrophoresis (CE) using the ZipChip Interface Ti (908 Devices) with a High Resolution (HR) Chip, ACE RM Background Electrolyte (BGE). The ZipChip Interface operated with a field strength of 500 V/cm, pressure assist on at 0 min, and an injection volume of 5.5 nL. The ZipChip Interface was coupled to the Orbitrap Fusion Lumos Tribrid Mass Spectrometer (Thermo Scientific, Waltham, MA, USA). The Orbitrap Fusion Lumos operated in positive ionization mode with a scan range of *m*/*z* 70–800, RF lens of 40%, resolution set to 60,000, and a normalized AGC target of 100%. MS2 scans were collected in data-dependent acquisition (DDA) mode with an exclusion duration of 2.5 s, a resolution of 15,000, and HCD stepped collision energies of 30, 50, and 150. Samples were analyzed in randomized order with QC samples analyzed at the beginning, middle, and end of analysis.

### 2.8. Metabolomics Data Analysis

System suitability tests and data checks were assessed for data quality (peak width, intensity, and resolution) prior to data processing in the ZipChip ACE Application (908 Devices). Data were processed in Skyline (version 24.1; MacCoss Lab, Universrity of Washington, Seattle, WA, USA; https://skyline.ms), and metabolites were identified based on accurate mass and indexed migration time. Automated peak integration was performed with a pre-release, Skyline-based software tool (908 Devices). The metabolomics analysis was considered semi-targeted because metabolites were quantified against a predefined panel of annotated metabolites using the capillary electrophoresis–mass spectrometry platform, rather than identified through an untargeted discovery workflow. Concentrations and normalized peak area ratios were calculated using the kit calibration standards and exported from Skyline. Data were imported into R and filtered with these conditions: (1) Individual values less than 3× the blank concentrations were excluded. (2) Molecules where relative standard deviations (%RSDs) measured in the SPQC samples (n = 3) were greater than 30% were excluded. (3) Molecules that were measured in less than 50% of the experimental samples were excluded. Unpaired, two-sided student’s *t*-tests were conducted in R. Further, a Benjamini–Hochberg false discovery rate (FDR) correction was additionally applied, and both raw *p*-values and FDR-adjusted q-values are reported. Missing values were imputed with 20% of the minimum observed value for each metabolite prior to log10 transformation, mean-centering and auto-scaling for PCA analysis with the prcomp package in R. Metabolite correlations are reported descriptively; 95% confidence intervals (Fisher’s z-transformation) for correlation heatmaps are provided in Table A3. Correlation analysis was done using R to generate Pearson correlation coefficients as well as heatmaps (pheatmap R package). Metabolites were considered to show a significant difference between groups if they met both a fold change of ≥1.2 or ≤0.8 (a 20% linear fold change) and a *p*-value < 0.05.

### 2.9. Proteomics Sample Preparation

For each sample, 12.5 µL of MagReSyn SAX beads (1:4 bead-to-plasma ratio) were washed twice with 200 µL of BTP wash buffer (50 mM Bis Tris Propane pH 6.3, 150 mM NaCl). A 50 µL subsample of each plasma sample was diluted 1:1 with bind buffer, supplemented with HALT inhibitors and mixed for 30 s. Diluted plasma was added to the equilibrated SAX beads and incubated with gentle mixing for 30 min in a thermomixer. Beads were then separated magnetically, the flow through removed, and washed 3x with BTP buffer.

For each sample, 100 µL lysis buffer and reduction mix (50mM Tris, pH 8.5, 1% SDS, 10 mM TCEP) were combined and incubated for 60 min at 37 °C with gentle mixing. Alkylation was performed by adding 15mM of iodoacetamide to each sample and incubating in the dark for 30 min with gentle mixing. Proteins were precipitated with the addition of 240 µL of acetonitrile (ACN) to each sample and incubated at RT for 10 min. Beads were washed 3 times with 500 µL of 95% ACN.

On-bead digestion was performed in 200 µL of 25mM ammonium bicarbonate with 0.5 µAU/µL LysC, with incubation at 47 °C for 2 h, followed by the addition of 4 µg of Trypsin (Promega, Madison, WI, USA) and incubation for 1.5 h at 47 °C with shaking. Digestion was quenched with 0.5% trifluoroacetic acid (TFA). Peptides were acidified to 0.5% TFA and desalted using Thermo desalting spin columns, dried by vacuum centrifugation, and quantified using Pierce Quantitative Fluorometric Assay. Samples were normalized to 0.2 µg/µL and subjected to LC-MS/MS analysis.

### 2.10. Proteomics LC-MS/MS

Samples were analyzed by LC-MS/MS using a Vanquish Neo coupled to an Orbitrap Astral mass spectrometer (Thermo Scientific). A pooled sample was analyzed at the beginning and end of the sequence. Samples were injected onto an IonOpticks Aurora series 3 C18 column (75 µm id × 15 cm, 1.6 µm particle size; IonOpticks, Melbourne, Australia) and separated over a 30 min period. The gradient for separation consisted of 2–30% mobile phase B at a 300 nL/min flow rate, where mobile phase A was 0.1% formic acid in water and mobile phase B consisted of 0.1% formic acid in 80% ACN. The Orbitrap Astral was operated in Data Dependent Acquisition (DDA) mode, in which the most intense precursors were selected within a 0.6 s cycle for subsequent HCD fragmentation and Astral MS/MS detection. For full MS scans, *m*/*z* was set to 375–1500, resolution was set to 180,000, maximum injection time was 5 ms, and the AGC target was 300%. MS/MS scans were acquired in the Astral analyzer using an *m*/*z* range of 110–2000, a maximum injection time of 2.5 ms, and an AGC target of 100%. The normalized collision energy was set to 30% for HCD, with an isolation window of 2 *m*/*z*. Precursors with unknown charge or a charge state of 1 and ≥7 were excluded.

### 2.11. Proteomics Data Analysis

Raw data files were processed using Spectronaut (v19; Biognosys, Schlieren, Switzerland) and searched against the Uniprot reviewed human database (UP000005640, containing 20,441 entries, downloaded January 2024). The following settings were used: enzyme specificity set to trypsin, up to two missed cleavages allowed, cysteine carbamidomethylation set as a fixed modification, methionine oxidation, and N-terminal acetylation set as variable modifications. We additionally applied a threshold of |log_2_FC| ≥ 0.6 (~1.5-fold) to better identify proteins that had larger differences between groups. A false discovery rate (FDR) of 1% was used to filter peptide spectrum match (PSM), peptide, and protein group identifications. Unpaired Student’s *t*-tests and Storey’s multiple test correction were conducted in Spectronaut. Molecules that were measured in less than 50% of the experimental samples were excluded. Unpaired *t*-tests were conducted in Spectronaut. Pearson correlations were calculated between metabolite intensities and anthropometric/physical function measures for both metabolomics and proteomics data separately. Protein correlation coefficients are similarly presented as descriptive given the exploratory design and limited sample size; corresponding 95% confidence intervals, calculated via Fisher’s z-transformation, are reported in Table A3. Correlation analysis was done using R to generate Pearson correlation coefficients as well as heatmaps (pheatmap R package). For correlations, proteins were excluded from analysis if <50% of participants showed detectable amounts of that protein. Proteins were filtered for |r| > 0.75 in at least one correlation and the top 5 most correlated anthropometric/physical function parameters were displayed in the heatmaps (both metabolomic and proteomic maps). Given the small subgroup sample sizes (n = 12 Pre-Frail, n = 6 Frail), Pearson correlation assumptions could not be reliably verified for each analyte pair, and individual values may exert disproportionate influence on the resulting correlations. These analyses should therefore be considered descriptive and exploratory. Where a difference in the direction of a correlation was observed between the Pre-Frail and Frail groups, we performed a formal statistical comparison of the two independent correlation coefficients using Fisher’s r-to-z transformation.

### 2.12. REAP-S-Specific Analysis

We conducted an item-level REAP-S analysis by frailty status, as well as correlation analyses between total REAP-S scores and our most prominent metabolomic marker: nicotinamide. Items were compared either using Mann–Whitney U or Fisher exact tests, where appropriate. Comparisons were not corrected for multiple testing given our small sample size and limited power of these corrections, and thus should be interpreted descriptively and as hypothesis-generating. Correlation analysis was done using Spearman rank correlation, given the ordinal arrangement of the REAP-S questionnaire.

## 3. Results

### 3.1. Baseline Characteristics

A total of n = 18 consecutive participants were assessed for eligibility; none were excluded. There were no robust participants classified using the CDI in our sample (Table 1). Plasma samples for multi-omics analysis were available for the entire cohort (n = 18 participants). One participant declined to participate in physical function survey measures (Table 2). Mean ± SD age was 77.1 ± 5.5 years (33% female), with a mean of 2.6 ± 2.0 comorbidities per participant in this highly educated cohort. Twelve (66.7%) were classified as pre-frail and 6 (33.3%) as frail using the CDI; there were no robust participants categorized. Baseline characteristics were generally similar between CDI-defined pre-frail and frail groups, with only a difference in sex distribution. Specifically, all 6 participants with frailty were male whereas the pre-frail group had 6 male participants and 6 female participants. Given the known differences in metabolism and body composition based on sex, this represents a potential confounding variable in our analysis. Stratifying by the Fried Index, there were 6 patients (33.3%) classified as robust, 7 (38.9%) as pre-frail, and 5 (27.8%) as frail. The groups differed at baseline with regard to age (*p* = 0.02), education (*p* = 0.04), and living conditions/support (*p* = 0.01) (Table A1).

### 3.2. Anthropometrics and Physical Function Based on Cumulative Deficit Frailty

There were no major differences in baseline demographic characteristics between participants classified as pre-frail vs. frail. However, at baseline, there was a significant difference in gait speed (*p* = 0.01), IADL impairments (*p* = 0.005), and CHAMPS score (*p* = 0.006) between pre-frail and frail participants. Because gait speed and IADL dependence are themselves scored components of the CDI, these two comparisons are not to be interpreted as independent of the exposure used to classify the groups. We do acknowledge that these strength and performance measures are often evaluated in the context of frailty assessments despite their inclusion in the aforementioned definitions.

### 3.3. Metabolomics/Proteomics

**Figure 1:**
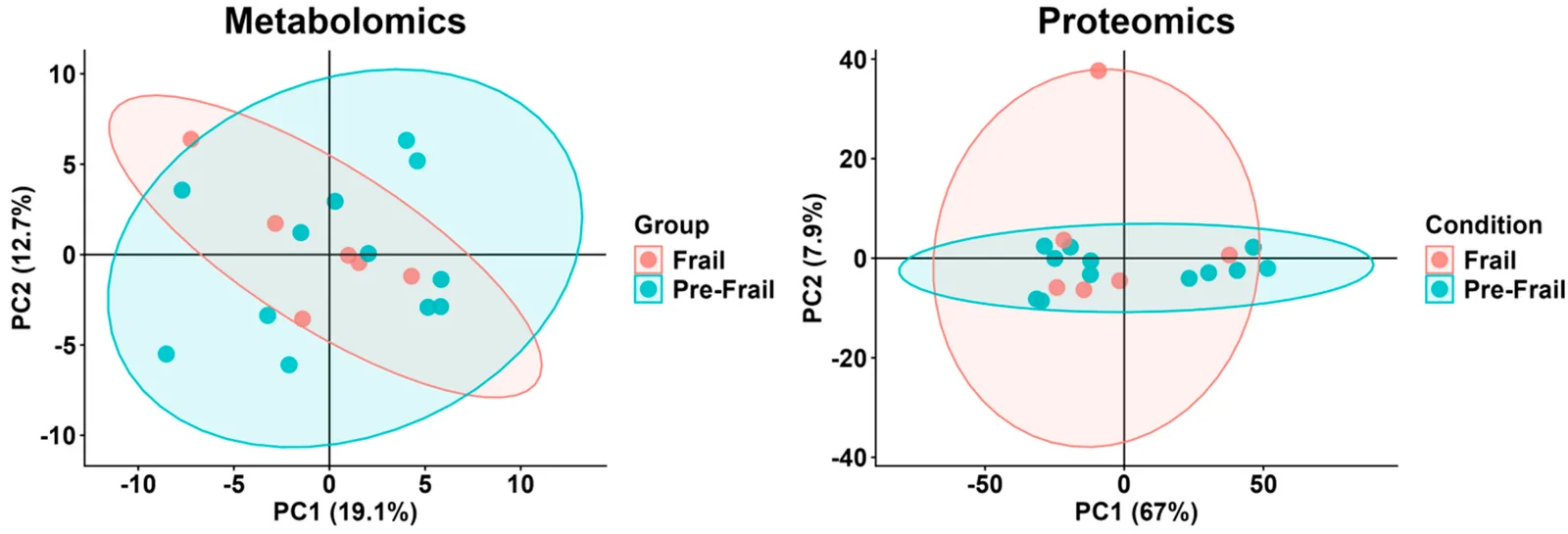
Metabolomics and proteomics principal component analysis plot for Cumulative Deficit Index (Frail vs. Pre-Frail). **Metabolomics**: Principal component analysis shows overlapping distributions of metabolite profiles between the cohorts with pre-frailty and frailty, indicating limited separation between groups along the primary components of variance. **Proteomics**: Principal component analysis similarly shows substantial overlap in protein profiles between the two groups.

**Figure 2:**
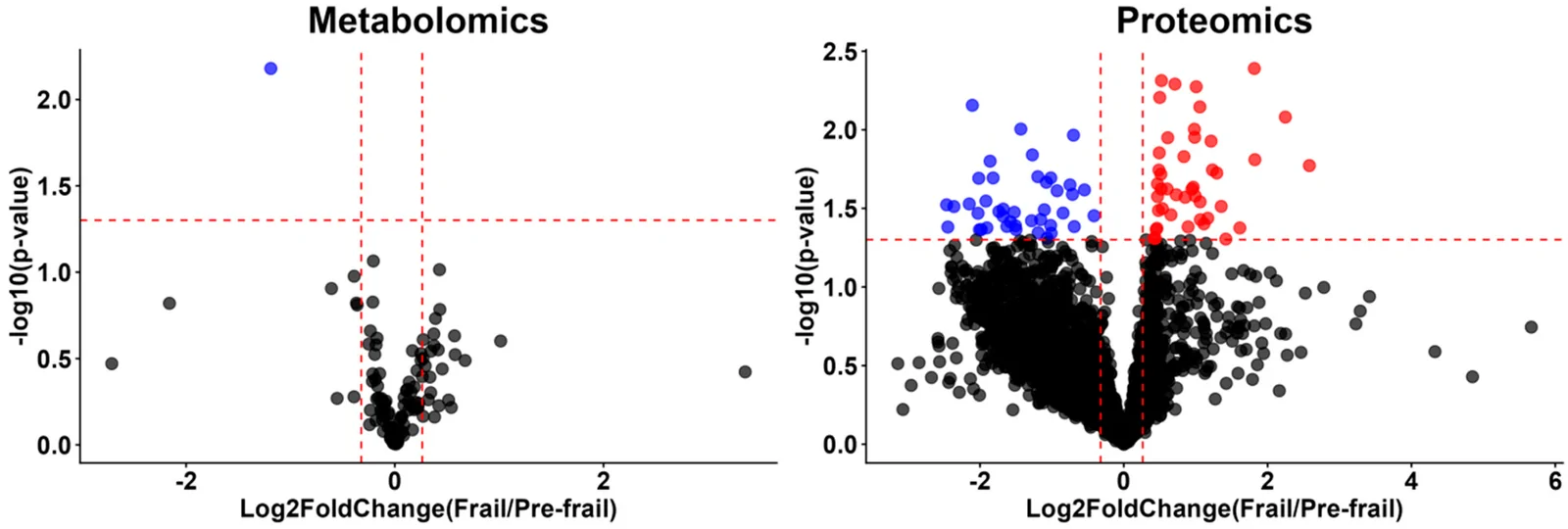
Metabolomics and proteomics volcano plots for Cumulative Deficit Index (Frail vs. Pre-Frail). **Metabolomics**: Volcano plot shows metabolites with a fold change ≥1.2 or ≤0.8 and a *p*-value < 0.05. Nicotinamide was the only metabolite meeting both criteria and was significantly decreased among participants with frailty; it did not remain significant after Benjamini–Hochberg FDR correction (q = 0.753) and is presented as an exploratory signal only. Other metabolites exceeded the fold-change threshold but did not reach statistical significance. **Proteomics**: Points in red indicate proteins that were significantly higher (right) or lower (left) in the group with frailty, based on FDR-adjusted q-values (Storey’s method) < 0.05 and a log_2_FC of ≥0.6 or ≤−0.6. Points in grey represent protein markers with an FDR-adjusted q-value < 0.05 but not meeting the log_2_FC of ≥0.6 or ≤−0.6.

**Figure 3:**
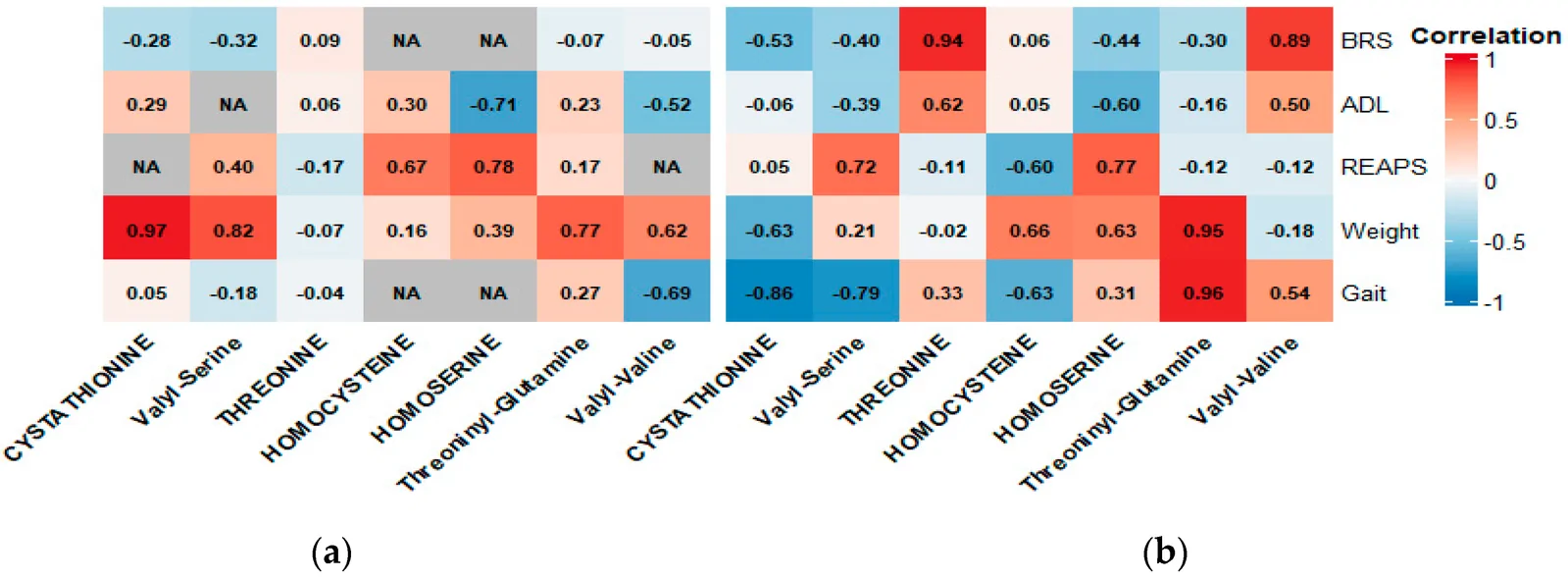
Pre-Frail and Frail metabolomic correlation heatmaps. Heatmap showing Pearson correlation coefficients between anthropometric and physical function measures (*y*-axis) and metabolomic markers (*x*-axis) in the cohorts with (**a**) pre-frailty (left), and (**b**) frailty (right). Darker color boxes indicate stronger correlations. Red indicates a positive or direct correlation while blue represents a negative or inverse correlation. Weight demonstrated consistent strong correlations with amino acids across both cohorts. Gait showed predominantly negative correlations among the cohort with frailty, with the exception of threoninyl-glutamine. Overall metabolomic correlation patterns were heterogenous and similar across the cohorts. Only analytes that meet our threshold of |r| > 0.75 are displayed.

Metabolomic analyses identified and measured 114 metabolites. The percent relative standard deviation (%RSD) was 7.7% in the SPQC sample, indicating high technical reproducibility. Using the CDI, the pre-frail and frail groups had %RSDs of 36.6% and 28.8%, respectively. A principal component analysis showed overlapping responses between the pre-frail and frail groups (Figure 1). Nicotinamide was decreased among the cohort with frailty compared to pre-frailty (*p* = 0.0066; log_2_FC = −1.19) but did not remain significant after Benjamini–Hochberg FDR correction (q = 0.753). Additionally, no metabolite retained significance after correction. We therefore present nicotinamide as an exploratory finding warranting further investigation, not a confirmed frailty-associated signal (Figure 2). In the metabolomic correlation analysis, the majority of highly correlated analytes were amino acids. Weight showed consistent strong, positive correlations with amino acid metabolites across both pre-frail and frail cohorts. Gait speed showed stronger correlations with amino acid metabolites among participants with frailty as opposed to pre-frailty. These frail correlations in gait speed were predominantly strong and negative, but threoninyl-glutamine was a notable strong positively correlated marker in this cohort. BRS showed strong positive correlations with threonine and valyl-valine in the cohort with frailty only. ADL showed heterogenous results with moderate positive and negative correlations among both cohorts (Figure 3). Overall, the correlation patterns were similar across cohorts, both showing mixed positive and negative correlations (Figure 3).

**Figure 4:**
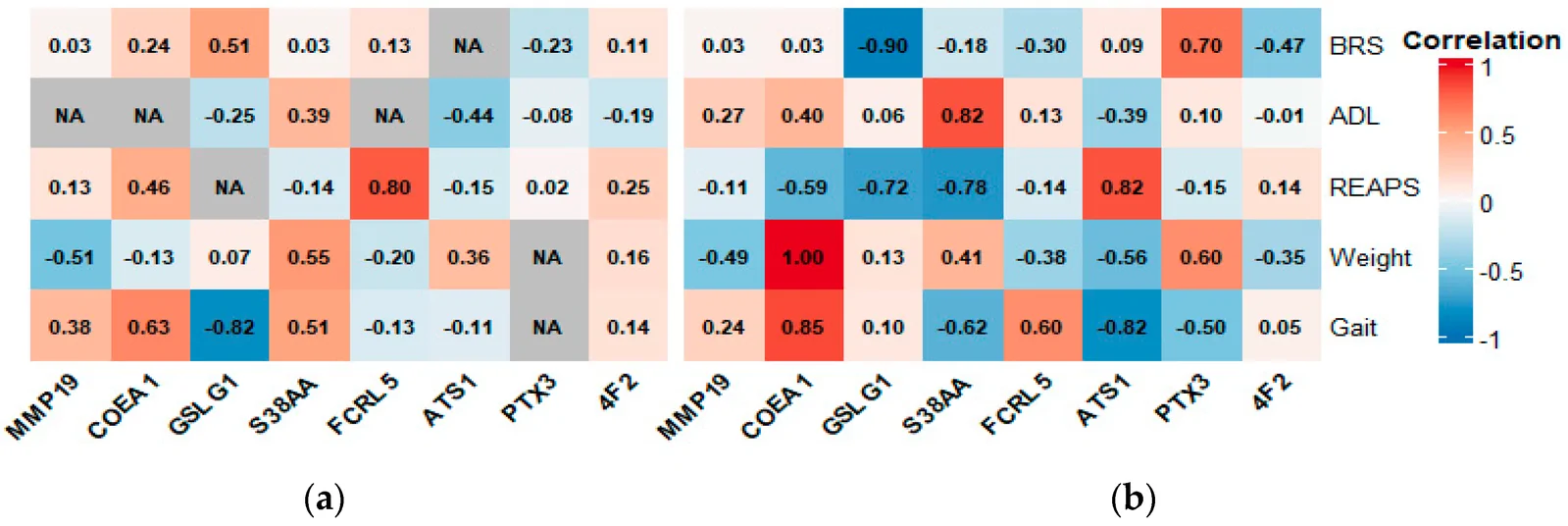
Pre-Frail and Frail proteomics correlation heatmaps. Heatmaps showing Pearson correlation coefficients between anthropometric/physical function measures (*y*-axis) and protein markers (*x*-axis) in participants with (**a**) pre-frailty (left) and (**b**) frailty (right). Correlations are colored by magnitude (darker = stronger) and direction (red = positive, blue = negative). For a subset of proteins, the sign of the correlation differed between groups; however, with n = 6–12 per group, these coefficients are imprecise (95% CIs in Table A4). Further, larger absolute correlations in the group with frailty may partly reflect its smaller sample size. Of the 18 apparent reversals in correlation direction, only 3 differed significantly between groups (Fisher r-to-z, *p* < 0.05). These patterns are descriptive and hypothesis-generating and should not be interpreted as evidence of a biological transition between frailty states.

In the proteomics analysis, over 4300 proteins were quantified. There was substantial overlap between frail and pre-frail participants (Figure 1). Sixty-five proteins met predefined differential thresholds between groups (FDR-adjusted q-value < 0.05, Storey’s method). Of these, 53 also had a |log_2_FC| ≥ 0.6, indicating larger differences between groups. Twenty-six of these 53 were higher among participants with frailty, and 27 were higher among participants with pre-frailty. The 26 proteins elevated among participants with frailty (log_2_FC > 0.6) included several histone proteins (H2B, H3C1, H2AC3, H3-4), as well proteins related to translation/muscle metabolism (Golgi apparatus protein 1, large ribosomal subunit protein mL65, troponin C) (Figure 2). In contrast, the 27 proteins higher in participants with pre-frailty (log_2_FC < −0.6) included proteins related to cell antioxidant defense (thioredoxin reductase 1 and glutathione S-Transferase Mu2) and the electron transport chain (NADH dehydrogenase) (Figure 2). In this exploratory, descriptive analysis, correlations between protein levels and physical function measures were generally stronger in the frail cohort than the pre-frail cohort, and the direction of most protein correlations differed between groups (more often positive in pre-frailty, negative in frailty). REAPS and gait speed showed the strongest correlations with protein biomarker levels among participants with frailty (Figure 4). Given the small sample size in each group, correlation estimates carried wide 95% confidence intervals (Table A3), and these patterns should be interpreted as descriptive and exploratory rather than as evidence of a biological transition between frailty states. Furthermore, correlation coefficients between protein levels and anthropometric/physical function measures differed in direction between the two cohorts for a subset of analytes. However, after Fisher r-to-z comparisons of independent correlation coefficients, only 3 of the 18 apparent reversals reached statistical significance (*p* < 0.05). This further underscores the limited power of this comparison given the sample sizes involved (n = 12 Pre-Frail, n = 6 Frail). Thus, these findings should be interpreted as descriptive and hypothesis-generating (Figure 4).

### 3.4. REAP-S Analysis

To characterize dietary patterns specifically across frailty status, we examined individual items from the REAP-S questionnaire. Total REAP-S score did not differ significantly between participants with frailty vs. pre-frailty (29.2 ± 5.8 vs. 28.6 ± 3.6; *p* = 0.83). Item-level analysis demonstrated that participants with frailty were less likely to report usually feeling well enough to shop or cook (2/5, 40%) compared to participants with pre-frailty (12/12, 100%; Fisher’s exact *p* = 0.015). No other individual REAP-S item differed significantly between groups (all *p* > 0.11). Exploratory correlation of total REAP-S score with nicotinamide, our primary metabolomic finding, showed a positive, non-significant trend (Spearman’s ρ = 0.37, *p* = 0.16).

## 4. Discussion

Our exploratory findings suggest several candidate metabolomic and proteomic markers that may warrant further investigation across the frailty spectrum. Given our cross-sectional design and small sample size, these should be interpreted as hypothesis-generating rather than as establishing a biological basis for frailty. Specifically, metabolomic and proteomic markers associated with amino acid metabolism, DNA—nucleosomes and histones—extracellular matrix remodeling, and inflammatory response differed between groups and may be worth exploring as candidate biomarkers in future, larger studies. However, because gait speed and IADL dependence are scored components of the CDI itself, these differences are expected and should not be interpreted as independent validation of the CDI’s classification ability. The CHAMPS physical activity difference, which is not a CDI component, more directly supports the CDI’s validity and is consistent with the previous literature associating frailty with reduced physical activity [8,35]. Further, the CDI and Fried Index classified our cohort differently, most notably in the robust category: no participants met CDI criteria for robust status, whereas six were classified as robust using the Fried phenotype. This discordance is consistent with the literature showing only low-to-moderate agreement between deficit-accumulation (CDI) and phenotypic measures (Fried) [36]. This pattern likely reflects how these two definitions construct frailty: the CDI combines deficits across a wide range of health domains and is sensitive to any accumulated burden, while the Fried phenotype captures a narrower, physical/functional presentation of frailty. In the context of our omics findings, the molecular signatures we identified should be interpreted with the CDI’s deficit-accumulation operationalization of frailty specifically, rather than with frailty as a whole.

Metabolomic markers are critically important as they may be associated with a di-verse array of complex syndromes, including frailty [25,37]. In our exploratory analysis, nicotinamide was nominally lower among participants with frailty, though this did not lead to significance after correction for multiple testing. This should be interpreted as a hypothesis-generating signal only, given our small sample size and cross-sectional design. One biologically sensible hypothesis is that reduced nicotinamide availability could limit NAD+-dependent histone deacetylation, potentially contributing to the altered histone protein levels we observed [38,39,40]. Whether this pathway is actually at play would require longitudinal or interventional data to confirm. Prior work has proposed that the NAD+ pathway may be relevant to muscle decline and mitochondrial dysfunction in frailty, which our findings are consistent with but do not confirm [39,41]. Furthermore, since our metabolomic data reflect plasma concentrations at a single timepoint, they do not directly reflect intracellular or tissue-level NAD+ availability, NAD+ metabolic flux, or sirtuin enzymatic activity, which would require specific assays that were not conducted in this study. In reference to the correlation results, the correlation patterns we observed across frailty phenotypes consistently involved amino acid metabolites, suggesting amino acid metabolism as an area worth further investigation in relation to frailty. Furthermore, weight was correlated with several of these amino acid metabolites. Together, we hypothesize that weight and amino acid metabolism may be interconnected in the context of frailty; however, our cross-sectional design cannot establish this relationship entirely. These findings are consistent with the prior literature linking alterations in amino acid metabolism with muscle mass, sarcopenia, and frailty [42,43,44].

Our proteomic analysis demonstrated that histone proteins, translational machinery, and muscle metabolism proteins were elevated among participants with frailty. Histone proteins are important for maintaining the structure and integrity of DNA, and elevated levels of these proteins may be associated with inflammatory issues, auto-immune conditions, and even cancer [45]. Furthermore, the elevated muscle metabolism proteins including troponin and translational machinery such as ribosomal and Golgi body subunits have been shown to be associated with oxidative stress and muscle damage [46,47,48]. Oxidative stress has been consistently proposed as a contributor to aging and functional decline [48,49,50]. However, while our cross-sectional data are consistent with this hypothesis, we cannot establish whether oxidative stress is a cause, consequence, or co-occurring feature of frailty in our cohort. Patterns of correlation between protein levels and physical function parameters differed between the pre-frail and frail groups. Because our design was cross-sectional and the sample size limited, we cannot determine whether these differences reflect true biological change over the course of frailty progression and are causal in nature, or are due to sampling variability, or both; the wide confidence intervals around individual correlation estimates (Table A4) highlight this uncertainty. However, the proteins most strongly correlated with physical function in our data are heavily involved in extracellular matrix remodeling, amino acid transport, and inflammatory signaling—pathways that are biologically plausible candidates for further study given the literature on ‘inflammaging’ and inflammatory dysregulation in frailty [51,52,53]. We view these correlation patterns as hypothesis-generating rather than confirmatory evidence of a frailty-related biological transition; longitudinal studies with larger samples will be needed to test whether these associations actually change as individuals progress through frailty states. Several of our candidate metabolomic markers—nicotinamide for instance—are largely obtained through dietary means (niacin- or tryptophan-derived protein sources). While our exploratory correlation between total REAP-S score and nicotinamide did not reach statistical significance, the direction of this association is consistent with a nutritional contribution to the metabolomic differences observed across frailty status and warrants targeted investigation with validated dietary intake instruments in future, adequately powered studies. Furthermore, participants with frailty were less likely to report functional stability when it comes to shopping/food preparation, suggesting that functional decline may be an important, underexplored pathway linking frailty to nutritional risk in this population. However, as this finding with the REAP-S questionnaire overlaps with the IADL survey and thus the CDI as a whole, it should be interpreted as hypothesis-generating rather than as an independent nutritional deficit.

While we maintain that our findings are exploratory, this study has several important strengths. First, we used two frailty indices to examine physical function in our cohort [6,8]. Third our methodology using high-throughput analyses of multi-omic profiling provides a broad and rigorous assessment of molecular features associated with frailty. Also, the study was conducted in a pragmatic, real-world setting, supporting the relevance of these findings to clinically representative populations. Concurrently, we acknowledge that our findings should be interpreted with the following limitations in mind. Primarily, our limited sample size prevents us from extending our inferences but provides an opportunity to conduct targeted analyses in the future to evaluate longitudinal changes in these markers [54]. Second, all participants with frailty in our cohort were male, whereas the group with pre-frailty was evenly split by sex. Although a sex-restricted sensitivity analysis showed that fold-changes for both metabolomics and proteomics closely tracked the full-sample results, this comparison was itself limited by small sample size (n = 6 per group); thus, confounding by sex cannot be fully excluded. Larger, sex-balanced cohorts will be needed to attenuate the independent contributions of sex to these molecular signatures. Normality and homogeneity of variance were also not formally assessed due to limited statistical power in our small sample size. Some variables (IADL) exhibited a ceiling effect (no variance with maximum scores), which inherently violates the assumptions made under a *t*-test. Results generated for such variables (*p*-values and effect sizes) should thus be interpreted with caution. Additionally, while our modified CDI has not been independently validated as a standalone tool, our CDI followed established guidance and robust methodology for accumulation-of-deficit frailty indices, as outlined by Searle et al. [55]. However, our deficit domains were not equally informative. Medication burden, scored using a graded threshold (0–5 points based on total medication count), contributed a mean of 27.7% (median 31.7%, range 0–55.6%) of individual participants’ total CDI scores, with 4 of 18 participants (22%) having medication account for at least half of their score. Additionally, comorbidity burden, drawn from a 16-item list, represented a similarly large potential share of the index (up to 16 of 37 points). Participants with greater polypharmacy or multimorbidity could therefore be classified as more frail based on these domains independent of physical function. This has been acknowledged as a limitation.

Our correlation and heatmap analyses comparing participants with frailty and pre-frailty are meant to be exploratory and descriptive in nature. With only 6–12 participants per group, correlation estimates carry substantial uncertainty (reflected in wide 95% confidence intervals; Table A3 and Table A4), and apparent differences in correlation magnitude or direction between groups should not be interpreted as evidence of meaningful biological transitions until further confirmation in, adequately powered, longitudinal cohort studies. Additionally, we acknowledge that the CDI and Fried Index classified our cohort differently—specifically that the Fried Index classified participants as robust whereas the CDI did not (Table 1 and Table A1). Furthermore, our omics analyses were restricted to the CDI classification of frailty. Despite broadly consistent findings for our anthropometric and physical function measures using the Fried Index and the CDI (Table A1 and Table A2), the CDI and Fried Index classified our cohort quite differently. The molecular patterns identified here should therefore be interpreted as specific to this CDI definition of frailty, and not generalized to frailty as defined by other indices or as a syndrome as a whole. Additionally, because this study is cross-sectional and exploratory with small group sample sizes, the associations we describe between metabolomic/proteomic markers and frailty status cannot establish causal or mechanistic pathways. Furthermore, the biological interpretations offered throughout this discussion should be read as hypotheses warranting testing in larger, independent, and longitudinal cohorts. In addition, while our proteomic results showed elevated plasma histone protein levels, these findings on their own cannot be interpreted as evidence of altered DNA integrity or epigenetic changes, given plasma histone’s sensitivity to both pre-analytical factors (e.g., hemolysis via processing) and non-specific biological processes unrelated to frailty. While samples were processed under standardized conditions within 60 min of collection, hemolysis was not specifically assessed as an exclusion criterion. Thus, to better identify DNA integrity or epigenetic regulation, specific assays are needed (e.g., cell-free DNA quantification, chromatin integrity assays, or direct epigenetic profiling).

Also, no participants in our cohort were classified as robust using the CDI; thus, our analyses are unable to characterize how these molecular profiles relate to a non-frail reference group. Participants were recruited from a single geriatric specialty clinic and were all referred by their clinical provider to a physical therapy program. The cohort was predominantly White (94%) and highly educated. Together, these factors limit the generalizability of our findings to broader, more diverse populations of older adults. We also were unable to adjust for dietary intake or body composition, both of which can meaningfully affect metabolomic and proteomic profiles independent of frailty status, as a result of our limited sample size. Similarly, we acknowledge that some measures that were included in the CDI classification included specific physical function outcome measures (as discussed in the Results and Discussion); hence, we caution the reader in the interpretation our findings. Finally, this was a single-cohort study without an independent replication or external validation cohort. The purpose was feasibility and to obtain variance estimates that could help power a future trial. Moreover, the molecular patterns identified here require confirmation in larger, more demographically diverse populations before they can be considered robust or generalizable markers of frailty. Additionally, we defined low physical activity for the Fried Frailty Index using the lowest quintile of the CHAMPS-calculated kcal/week expenditure among our sample, as opposed to using a previously validated population cutoff. Given our small sample size (n = 18), our sample-dependent cutoff is considerably vulnerable to instability, and thus should be interpreted as such—a relative ranking within our cohort, not an externally validated classification of low physical activity. Lastly, the correlation analyses in this study relied on Pearson correlation coefficients, which assume a linear relationship between variables and are sensitive to outlier observations. Given the small subgroup sizes, these assumptions could not be rigorously verified for each analyte pair, and a small number of extreme or atypical values may have disproportionately influenced individual correlation estimates. Non-parametric alternatives such as Spearman rank correlation, or resampling-based approaches such as bootstrap confidence intervals may better characterize the uncertainty around these estimates in future work. We did not re-analyze the current dataset using these methods, as the same small sample sizes limit the power of any correlation. Future studies with larger, adequately powered cohorts should consider Spearman correlation and bootstrap resampling as more robust approaches to characterizing these relationships, alongside formal comparison of correlation coefficients across subgroups when directional differences are of interest.

## 5. Conclusions

This study suggests that DNA/histone-associated markers and nicotinamide—a precursor in NAD+ metabolism—may be associated with frailty. In addition, amino acid metabolites, extracellular matrix remodeling complexes, and inflammatory mediators warrant further investigation as potential contributors to frailty biology. We propose that a future targeted analysis in independent cohorts will be important to validate these preliminary findings and determine whether these markers can identify individuals at increased risk for frailty progression. We also hope further research explores, in more detail, these classes of biomarkers (i.e., extracellular matrix remodeling, amino acid, and inflammatory signaling). These results may also help inform future randomized trials evaluating whether lifestyle interventions, such as physical therapy or exercise, or medical treatment strategies can modify molecular markers associated with frailty.

## Figures and Tables

**Table 1:** Baseline characteristics by cumulative deficit status of frailty.

Characteristics	Total	Pre-Frail **	Frail	*p* Value
	n = 18	n = 12	n = 6	
**Age, years**	77.1 ± 5.5	76.2 ± 6.3	79.2 ± 2.2	0.16
**Female Sex**	6 (33)	6 (50)	0 (0)	0.007
**Race**				0.34
White	17 (94)	11 (92)	6 (100)	
Black or African American	0 (0)	0 (0)	0 (0)	
Asian	1 (6)	1 (8)	0 (0)	
**Ethnicity (Hispanic/Latino)**	2 (11)	2 (17)	0 (0)	0.17
**Marital Status**				0.29
Married/Living with partner	12 (67)	9 (75)	3 (50)	
Widowed	3 (17)	2 (17)	1 (17)	
Separated, Divorced, Single	3 (17)	1 (8)	2 (33)	
**Health Insurance**				0.38
Medicare	18 (100)	11 (92)	6 (100)	
Medicaid	0 (0)	0 (0)	0 (0)	
Private	3 (17)	1 (8)	2 (33)	
**Smoking Status**				0.22
Non-smoker	13 (72)	10 (83)	3 (50)	
Former smoker	5 (28)	2 (17)	3 (50)	
Alcohol Use *				0.57
None	7 (41)	5 (42)	2 (40)	
1–5 Drinks/Week	4 (24)	2 (17)	2 (40)	
6+ Drinks/Week	6 (33)	5 (42)	1 (20)	
**Education** *				0.53
<High School	0 (0)	0 (0)	0 (0)	
High School Graduate or GED	1 (6)	1 (8)	0 (0)	
Some College	1 (6)	0 (0)	1 (20)	
College or Graduate Degree	15 (88)	11 (92)	4 (80)	
Household Annual Income *				0.74
<$25,000	2 (12)	0 (0)	2 (40)	
$25,000 to $49,999	3 (18)	3 (25)	0 (0)	
$50,000 to $74,999	1 (6)	1 (8)	0 (0)	
$75,000 to $99,999	2 (12)	2 (17)	0 (0)	
$100,000 to $199,999	7 (41)	5 (42)	2 (40)	
≥$200,000	2 (12)	1 (8)	1 (20)	
**Living Conditions/Support**				0.09
Own Home with No Home Support	15 (83)	12 (100)	3 (50)	
Home of Friend or Family Member	1 (6)	0 (0)	1 (17)	
Independent Living Facility	1 (6)	0 (0)	1 (17)	
Assisted Living Facility	1 (6)	0 (0)	1 (17)	
People Living with Participant				0.08
No One/Live Alone	3 (17)	3 (25)	0 (0)	
Spouse/Significant Other	13 (72)	9 (75)	4 (67)	
Friend(s)	2 (11)	0 (0)	2 (33)	
**Specific List of Comorbidities**				0.22
Anxiety	5 (28)	1 (8)	4 (67)	
Cardiovascular Disease	4 (22)	1 (8)	3 (50)	
Cognitive Impairment	5 (28)	0 (0)	5 (83)	
Depression	4 (22)	2 (17)	2 (33)	
Diabetes	2 (11)	1 (8)	1 (17)	
High Cholesterol	7 (39)	2 (17)	6 (100)	
Hypertension	9 (50)	5 (42)	4 (67)	
Osteoarthritis	5 (28)	3 (25)	2 (33)	
Sleep Apnea	3 (17)	0 (0)	3 (50)	

All values are represented as mean ± standard deviation or count (%). % values that sum >100% indicate some participants fit multiple criteria; PT: physical therapy at least once a week. * Items that do not add to 18 indicate a participant chose not to answer a question. Frailty stratification based on CDI. ** Cumulative deficit calculated by establishing a binary variable for 37 different indicators of frailty. For each participant, all index scores were summed and divided by 37: <0.08 = robust; 0.08–0.24 = pre-frail; >0.25 = frail.

**Table 2:** Baseline objective and contextual measures for Pre-Frail vs. Frail participants (CDI).

Objective Measures	Pre-Frail (n = 12)	Frail (n = 6)	*p* Value	Effect Size ^a^
Weight, kg	78.4 ± 15.5	82.3 ± 10.9	0.54	0.28
Grip Strength, kg	28.8 ± 9.5	28.7 ± 4.1	0.98	−0.01
Gait Speed, m/s	1.23 ± 0.26	0.81 ± 0.29	0.01	−1.55
ADL Score *	50.0 ± 0.0	45.0 ± 7.7	0.17	−1.15
IADL **	100.0 ± 0.0	60.7 ± 20.1	0.005	−3.50
**Subjective Measures**	**Pre-Frail** (n = 12)	**Frail** (n = 5) ^b^	***p*** **value**	**Effect Size**
CHAMPS, hours/wk ^c^	12.2 ± 9.9	2.25 ± 2.05	0.006	−1.16
Brief Resilience Scale ***	3.06 ± 0.28	3.00 ± 0.30	0.74	−0.19
REAP-S	28.6 ± 3.6	29.2 ± 5.8	0.83	0.14
PAM-13	87.7 ± 8.1	65.4 ± 18.9	0.06	−1.86
PFS	11.8 ± 7.9	22.4 ± 9.4	0.07	1.27

Values presented as mean ± SD; n (%); frailty classified based on the CDI. ^a^ Cohen’s d effect size, calculated as (frail group mean − pre-frail group mean)/pooled SD. Negative values indicate lower values among participants with frailty compared to participants with pre-frailty, and positive values indicate higher values among participants with frailty compared to pre-frailty. 95% confidence intervals were calculated per Hedges and Olkin (1985) [34]. Small effect = 0.2; medium effect = 0.5; large effect = ≥0.8. ^b^ One participant did not complete all subjective measures. ^c^ CHAMPS score presented as number of hours per week participants spent participating in moderate exercise activity including dance, golf, singles/doubles tennis, skating, heavy housework, heavy gardening, car work, jog/run, walking uphill, walking fast/briskly, riding a bicycle, other aerobic machines (rowing, stepping, etc.), water exercise, swimming (any intensity), aerobics, light, moderate, heavy strength training, basketball, soccer, or racquetball. Abbreviations: Activities of Daily Living (ADL), Instrumental Activities of Daily Living (IADL), Community Healthy Activities Model Program for Seniors (CHAMPS), Rapid Eating Assessment (REAP-S), Patient Activation Measure (PAM). * Calculated on a 0–50 scale with 50 being the most functional independence; 5-question survey assessing ability of participant to independently perform activities of bathing, dressing, toileting, ambulation, and feeding. Performing one activity with complete independence was awarded a score of 10. Ability to perform all activities independently equates to a total score of 50. This was modified from the Katz Index of Independence in Activities of Daily Living. ** Calculated as percent functionality with 100% functionality meaning completely independent. This is a 7-question survey assessing ability to independently perform activities of using the telephone, performing housework, managing finances, managing medication, shopping, preparing meals, and using transportation. A score of 0–2 was given for each activity with 0 equating to complete dependence, 1 meaning assistance is required, and 2 indicating complete independence. The total score was generated by summing all variables and dividing by the maximum possible score (14), to give a percent functionality. *** Higher score correlates with more positive outcomes.

## Data Availability

Data is available upon author request due to privacy.

## Abbreviations

The following abbreviations are used in this manuscript:

ADL	Katz Activities of Daily Living
CHAMPS	Community Health Activities Model Program for Seniors Physical Activity Scale
CDI	Cumulative Deficit Index
IADL	Lawson Instrumental Activities of Daily Living
LC-MS/MS	Liquid Chromatography-Tandem Mass Spectrometry
MET	Metabolic Equivalent Task
PAM-13	Patient Activation Measure (13-Item)
PFS	Pittsburgh Fatiguability Scale
REAP-S	Rapid Eating Assessment
SPQC	Study Pooled Quality Control

## Appendix A

### Appendix A.1. Description of Subjective Surveys

#### Appendix A.1.1. Activities of Daily Living

The Katz ADL Index assessed participants’ ability to independently perform activities of daily living: bathing, dressing, toileting, ambulation, continence, and feeding. Each response is given a score of either 0 (dependent), 5 (partially independent) or 10 (independent). For the purposes of this study, continence was not assessed. All responses were summed to give a possible score ranging from 0 (complete dependence) to 10 (complete independence). The 7-question Lawton IADL assessed an individual’s independent ability to perform living skills that are more complex than ADLs, including an ability to use the telephone, shop, prepare food, housekeep, engage in transportation, handle medications, and handle finances. Each response was given a score of either 0 (dependency), 1 (partial independence) or 2 (complete independence), for a max score of 14 per participant, with aggregate scoring presented as a percentage ranging from 0% (complete dependence) to 100% (complete independence).

#### Appendix A.1.2. Pittsburgh Fatigability Scale

The 13-item PFS is a valid, reliable, self-administered, 1-page assessment of perceived fatigability that was added to the BLSA in March 2011. The 10 items cover sedentary, social, lifestyle, and physical activities ranging from light to high intensity. The form instructs respondents to indicate the level of “physical and mental fatigue you expect or imagine you would feel immediately after completing each … activity” and to “please circle responses for both physical and mental fatigue between 0 and 5, where “0” equals no fatigue at all and “5” equals extreme fatigue.” Continuous scores for each dimension can range from 0 to 50, with higher scores indicating higher fatigability. Higher physical fatigability has been defined as a PFS-physical score of 15 and greater and higher mental fatigability as a PFS-mental score of 13 and greater. For this article, we also evaluated categorical scoring ranging from 0 to 5 for each scale. For the physical scale, 0 was assigned to scores of 0 to 4, 1 to scores of 5 to 9, 2 to scores of 10 to 14, 3 to scores of 15 to 19, 4 to scores of 20 to 24, and 5 to scores of 25 and higher. For the mental scale, 0 was assigned to scores of 0 to 3, 1 to scores of 4 to 7, 2 to scores of 8 to 11, 3 to scores of 12 to 15, 4 to scores of 16 to 19, and 5 to scores of 20 and higher.

#### Appendix A.1.3. Brief Resilience Scale

Scoring: The responses varying from 1–5 for all six items were added, giving a range of 6–30. The total sum was divided by the total number of questions answered. A higher score corresponds to more resistance.

#### Appendix A.1.4. REAP-S

The REAP-S is a 16-item questionnaire that assesses the dietary quality of the respondent and has been previously validated for use in various populations [6]. The first 13 items of the assessment inquire about various eating practices, including skipping breakfast, consuming food from restaurants, consuming less than recommended servings of whole grains, fruit, vegetables, dairy, and more than the recommended servings of meat, as well as consuming foods/drinks that are high in fats and sugars. These items are rated on a three-point frequency scale of “usually/often” (score = 1), “sometimes” (score = 2), and “rarely/never” (score = 3), with some items allowing for a “not applicable” (score = 0) response. Additionally, the survey asks two questions about shopping and cooking and one item inquiring about willingness to change eating habits. For a total instrument score, the first 13 items are summed for a total score ranging from 13 (low dietary quality) to 39 (higher dietary quality). The remaining three questions were analyzed separately.

#### Appendix A.1.5. PAM-13

PAM-13 has 13 items with a 5-point Likert response scale. The raw scores are summed and transformed to 0–100 metric (0 = lowest activation level, 100 = highest). Activation Level 1 (PAM Score < 47): Patients tend to be overwhelmed and unprepared to play an active role in their own health. Activation Level 2 (PAM Score 47.1–55.1): Patients lack knowledge and confidence for self-management. Activation Level 3 (PAM Score 55.2–67): Patients are beginning to take action, but lack confidence and skill to support behaviors. Activation Level 4 (PAM Score > 67): People have adopted many of the behaviors to support their health, but may not be able to maintain them in the face of life stressors.

#### Appendix A.1.6. CHAMPS Kcal/Wk Expended Calculation

Moderate intensity activities were the only ones assessed (MET ≥ 3.0). These activities included dance, golf, singles/doubles tennis, skating, heavy housework, heavy gardening, car work, jog/run, walking uphill, walking fast/briskly, riding a bicycle, other aerobic machines (rowing, stepping, etc.), water exercise, swimming (any intensity), aerobics, strength training (light, moderate, heavy), basketball, soccer, or racquetball. First, the frequency at which each moderate intensity activity was performed each week was calculated. We then calculated the hours per week that each moderate intensity activity was performed. A weighted duration variable was then calculated by multiplying the hours per week variable by the corresponding MET value for that activity. To get the number of kcals burned per week the weighted duration variable was multiplied by 3.5 and by 60 (converting METs/minute to METs/hour). Finally, this value was multiplied by weight (in kg/200). Then all kcal/wk values were summed from all the moderate intensity exercises to create a cumulative value of kcals burned per week doing moderate intensity exercise.

### Appendix A.2. Frailty Variable Definitions

#### Appendix A.2.1. Fried Frailty

Frailty was additionally classified using a modified Fried Frailty Phenotype [6]. Modifications to the original Fried criteria are common and well documented in the literature [56]. Exhaustion was measured using an item from the Pittsburgh Fatigability Scale (PFS), a validated measure of perceived fatigability in older adults, by asking the patient their physical fatigue after leisurely walking for 30 min. Any reporting of fatigue was given a score of 1 [57]. Gait speed <0.8 m/s was given a score of 1. This fixed threshold is used in place of the original sex- and height-stratified quintile approach, which has been recommended in the revised European consensus criteria for sarcopenia (EWGSOP2) [58]. Grip strength <35.5 kg and <20 kg for males and females, respectively, earned a score of 1. These fixed, sex-specific cutoffs are used in place of cutoffs stratified by sex and BMI as in the original phenotype, which is consistent with recent work finding grip strength correlates less strongly with BMI in an older adult cohort [59,60]. Further, our cutoffs are drawn from the Sarcopenia Definitions and Outcomes Consortium (SDOC), a more contemporary muscle-weakness classification derived from pooled analyses of multiple large cohort studies (Bhasin et al., 2020) [61]. Lastly, weight loss of >5% of starting body weight in the prior year was given a score of 1, consistent with the original Fried criterion [6]. Physical activity was assessed using the CHAMPS Questionnaire. For any activity deemed “moderate intensity” by CHAMPS (MET ≥ 3), the calories (kcal) burned per week doing these exercises were summed for each patient. Calories (kcal) burned per week was calculated by first creating an hours-per-week variable for each moderate intensity activity. A weighted duration variable was then calculated for each moderate intensity activity by multiplying the hours-per-week variable by the activity’s corresponding MET value. Calories expended per week for each activity was calculated by multiplying the weighted duration variable by 3.5 and by 60 (converting METs/minute to METs/hour and then by weight (in kg/200). The calorie expenditures per week for each moderate intensity activity were then summed, which gave the total calories expended per week doing moderate intensity activity (Appendix A.1). The lowest quintile scores for kcals burned per week were given a score of 1 (i.e., low physical activity).

#### Appendix A.2.2. Cumulative Deficit Frailty

One point was assigned for each of the following 37 deficits if the stated criteria were met. Body Mass Index (BMI) is an anthropometric based on weight and height; this received 1 point (a deficit) if >30 kg/m^2^. Depression screening was done using the PHQ-2 self-report questionnaire; one point was given if the score was ≥3. Resilience was measured using the BRS self-report questionnaire and was given one point if Question 1 (ability to bounce back quickly) was scored as a 1, 2, or 3 (indicating disagreement with being able to bounce back after hard times). Cognition was assessed using the SLUMS clinician-administered exam and received one point if score <24 (possible cognitive impairment). Fatigue was assessed using the PFS self-report questionnaire and received a point if Question 8 (participating in a social activity causes fatigue) is reported >0 (indicating any level of fatigue low–extreme). Gait speed is a timed physical performance test, and one point was given for a time <0.8 m/s. Grip strength was assessed using a dynamometer and was awarded one point if <35.5 kg (male) or <20 kg (female). Alcohol use was assessed via self-report and given one point for ≥3 drinks/week. ADL and functional dependence were given one point if any functional dependence was noted (score > 0) on feeding, dressing, ambulation, toileting, and bathing ADL items (one point given for each question). IADL dependence was given one point if any functional dependence (score > 0) was given for phone, shopping, meal, medication, finances, housework, and transportation IADL items (one point for each question). Each of the following 16 comorbidities were given one point if present: anxiety, cardiovascular disease, cognitive impairment, COPD, depression, diabetes, fibromyalgia, high cholesterol, hypertension, non-skin cancer, osteoarthritis, rheumatologic disease, sleep apnea, stroke, TIA, or other. Lastly, medicines were given one point if taking 5–7 and an additional point was given for every 3 medications taken.

**Table A1:** Baseline Characteristics by Fried Frailty Status (n = 18).

Characteristics	Total (n = 18)	Robust (n = 6)	Pre-Frail (n = 7)	Frail (n = 5)	*p* Value
**Age, years**	77.1 ± 5.5	72.8 ± 5.3	80.9 ± 3.2	76.8 ± 5.1	0.02
**Female Sex**	6 (33)	4 (67)	1 (14)	1 (20)	0.11
**Race**					0.29
White	17 (94)	6 (100)	7 (100)	4 (80)	
Black or African American	0 (0)	0 (0)	0 (0)	0 (0)	
Asian	1 (6)	0 (0)	0 (0)	1 (20)	
**Ethnicity (Hispanic/Latino)**	2 (11)	1 (17)	0 (0)	1 (20)	0.53
**Marital Status**					0.07
Married/Living with partner	12 (67)	5 (83)	6 (86)	1 (20)	
Widowed	3 (17)	0 (0)	1 (14)	2 (40)	
Separated, Divorced, Single	3 (17)	1 (17)	0 (0)	2 (40)	
**Health Insurance**					0.99
Medicare	18 (95)	6 (100)	6 (86)	5 (100)	
Medicaid	0 (0)	0 (0)	0 (0)	0 (0)	
Private	3 (17)	1 (17)	1 (14)	1 (20)	
**Smoking Status**					0.62
Non-smoker	13 (72)	4 (67)	6 (86)	3 (60)	
Former smoker	5 (28)	2 (33)	1 (14)	2 (40)	
**Alcohol Use ***					0.24
None	7 (41)	2 (33)	2 (29)	3 (75)	
1–5 Drinks/Week	4 (24)	2 (33)	1 (14)	1 (25)	
6+ Drinks/Week	6 (33)	2 (33)	4 (57)	0 (0)	
**Education** *					0.04
<High School	0 (0)	0 (0)	0 (0)	0 (0)	
High School Graduate or GED	1 (6)	1 (17)	0 (0)	0 (0)	
Some College	1 (6)	0 (0)	0 (0)	1 (25)	
College or Graduate Degree	15 (88)	5 (83)	7 (100)	3 (75)	
**Household Annual Income ***					0.08
<$25,000	2 (12)	0 (0)	0 (0)	2 (50)	
$25,000 to $49,999	3 (18)	1 (17)	1 (14)	1 (25)	
$50,000 to $74,999	1 (6)	0 (0)	1 (14)	0 (0)	
$75,000 to $99,999	2 (12)	1 (17)	1 (14)	0 (0)	
$100,000 to $199,999	7 (41)	3 (50)	3 (43)	1 (25)	
≥$200,000	2 (12)	1 (17)	1 (14)	0 (0)	
**Living Conditions/Support**					0.01
Own Home with No Home Support	15 (83)	6 (100)	7 (100)	2 (40)	
Home of Friend or Family Member	1 (6)	0 (0)	0 (0)	1 (20)	
Independent Living Facility	1 (6)	0 (0)	0 (0)	1 (20)	
Assisted Living Facility	1 (6)	0 (0)	0 (0)	1 (20)	
People Living with Participant					0.21
No One/Live Alone	3 (17)	1 (17)	1 (14)	1 (20)	
Spouse/Significant Other	13 (72)	5 (83)	6 (86)	2 (40)	
Friend(s)	2 (11)	0 (0)	0 (0)	2 (40)	
**Specific list of comorbidities**					0.17
Anxiety	5 (28)	0 (0)	1 (14)	4 (80)	
Cardiovascular Disease	4 (22)	0 (0)	2 (29)	2 (40)	
Cognitive Impairment	5 (28)	0 (0)	1 (14)	4 (80)	
Depression	4 (22)	2 (33)	0 (0)	2 (40)	
Diabetes	2 (11)	0 (0)	1 (14)	1 (20)	
High Cholesterol	7 (39)	2 (33)	2 (29)	3 (60)	
Hypertension	9 (50)	4 (67)	3 (43)	2 (40)	
Osteoarthritis	5 (28)	2 (33)	1 (14)	2 (40)	
Sleep Apnea	3 (17)	0 (0)	1 (14)	2 (40)	

* One participant was excluded due to providing no information.

**Table A2:** Baseline Objective and Contextual Measures by Fried Frailty Index.

Objective Measures	Robust (n = 6)	Pre-Frail (n = 7)	Frail (n = 5)	*p*-Value	Effect Size
Weight, kg	78.8 ± 14.0	80.3 ± 16.9	79.7 ± 12.0	0.98	−0.12
Grip Strength, kg	31.9 ± 10.8	28.4 ± 6.1	25.4 ± 5.9	0.42	−0.01
Gait Speed, m/s	1.20 ± 0.27	1.21 ± 0.28	0.77 ± 0.30	0.03	0.28
ADL Score	50.0 ± 0.0	50.0 ± 0.0	44.0 ± 8.2	0.052	0.22
IADL	100.0 ± 0.0	94.9 ± 13.5	60.0 ± 22.4	<0.001	0.56
**Subjective Measures**	**Robust (n = 6)**	**Pre-Frail (n = 7)**	**Frail (n = 4)**	***p*** **-value**	**Effect Size**
CHAMPS, h/wk	15.7 ± 11.1	8.5 ± 7.0	0.9 ± 1.9	0.04	0.26
Brief Resilience Scale	2.94 ± 0.23	2.98 ± 0.26	3.29 ± 0.29	0.11	0.16
REAP-S	28.5 ± 2.8	30.4 ± 3.5	26.3 ± 6.4	0.29	0.04
PAM-13	84.6 ± 8.0	87.6 ± 13.7	64.4 ± 17.9	0.03	0.28
Pittsburgh Fatigability	7.00 ± 2.28	13.9 ± 4.9	28.8 ± 5.9	<0.001	0.77

Values presented as mean ± SD; n (%). Frailty classified based on the CDI. Abbreviations: Activities of Daily Living (ADL), Instrumental Activities of Daily Living (IADL), Community Healthy Activities Model Program for Seniors (CHAMPS), Rapid Eating Assessment (REAP-s), Patient Activation Measure (PAM). See Table 2 and Appendix A for detailed descriptions of each measure.

**Table A3:** 95% Confidence Intervals for Correlations Between Metabolite Abundance and Physical Function/Anthropometric Measures, by Frailty Status.

Anthropometric Measure	Analyte	n (Pre-Frail)	r (95% CI) Pre-Frail	n (Frail)	r (95% CI) Frail
BRS	CYSTATHIONINE	12	−0.28 (−0.74 to 0.35)	6	−0.53 (−0.94 to 0.50)
BRS	Valyl-Serine	12	−0.32 (−0.75 to 0.31)	6	−0.40 (−0.91 to 0.61)
BRS	THREONINE	12	0.09 (−0.51 to 0.63)	5	0.94 (0.31 to 1.00)
BRS	HOMOCYSTEINE	12	NA	6	0.06 (−0.79 to 0.83)
BRS	HOMOSERINE	12	NA	6	−0.44 (−0.92 to 0.58)
BRS	Threoninyl-Glutamine	12	−0.07 (−0.62 to 0.52)	5	−0.30 (−0.93 to 0.79)
BRS	Valyl-Valine	12	−0.05 (−0.60 to 0.54)	6	0.89 (0.27 to 0.99)
ADL	CYSTATHIONINE	12	0.29 (−0.34 to 0.74)	6	−0.06 (−0.83 to 0.79)
ADL	Valyl-Serine	12	NA	6	−0.39 (−0.91 to 0.62)
ADL	THREONINE	12	0.06 (−0.54 to 0.61)	6	0.62 (−0.38 to 0.95)
ADL	HOMOCYSTEINE	12	0.30 (−0.33 to 0.75)	5	0.05 (−0.87 to 0.89)
ADL	HOMOSERINE	12	−0.71 (−0.91 to −0.23)	5	−0.60 (−0.97 to 0.60)
ADL	Threoninyl-Glutamine	12	0.23 (−0.40 to 0.71)	6	−0.16 (−0.86 to 0.75)
ADL	Valyl-Valine	12	−0.52 (−0.84 to 0.08)	6	0.50 (−0.52 to 0.93)
REAPS	CYSTATHIONINE	12	NA	6	0.05 (−0.79 to 0.83)
REAPS	Valyl-Serine	12	0.40 (−0.22 to 0.79)	5	0.72 (−0.44 to 0.98)
REAPS	THREONINE	12	−0.17 (−0.68 to 0.44)	6	−0.11 (−0.85 to 0.77)
REAPS	HOMOCYSTEINE	12	0.67 (0.15 to 0.90)	5	−0.60 (−0.97 to 0.60)
REAPS	HOMOSERINE	12	0.78 (0.37 to 0.93)	5	0.77 (−0.35 to 0.98)
REAPS	Threoninyl-Glutamine	12	0.17 (−0.44 to 0.68)	6	−0.12 (−0.85 to 0.77)
REAPS	Valyl-Valine	12	NA	6	−0.12 (−0.85 to 0.77)
Weight	CYSTATHIONINE	7	0.97 (0.79 to 1.00)	3	−0.63 (NA)
Weight	Valyl-Serine	7	0.82 (0.19 to 0.97)	5	0.21 (−0.82 to 0.92)
Weight	THREONINE	10	−0.07 (−0.67 to 0.58)	3	−0.02 (NA)
Weight	HOMOCYSTEINE	8	0.16 (−0.62 to 0.78)	4	0.66 (−0.82 to 0.99)
Weight	HOMOSERINE	7	0.39 (−0.51 to 0.88)	4	0.63 (−0.84 to 0.99)
Weight	Threoninyl-Glutamine	6	0.77 (−0.11 to 0.97)	3	0.95 (NA)
Weight	Valyl-Valine	6	0.62 (−0.39 to 0.95)	3	−0.18 (NA)
Gait	CYSTATHIONINE	12	0.05 (−0.54 to 0.61)	5	−0.86 (−0.99 to 0.09)
Gait	Valyl-Serine	12	−0.18 (−0.68 to 0.44)	6	−0.79 (−0.98 to 0.07)
Gait	THREONINE	12	−0.04 (−0.60 to 0.55)	5	0.33 (−0.78 to 0.94)
Gait	HOMOCYSTEINE	12	NA	6	−0.63 (−0.95 to 0.37)
Gait	HOMOSERINE	12	NA	6	0.31 (−0.67 to 0.90)
Gait	Threoninyl-Glutamine	12	0.27 (−0.36 to 0.73)	5	0.96 (0.46 to 1.00)
Gait	Valyl-Valine	12	−0.69 (−0.90 to −0.19)	5	0.54 (−0.65 to 0.96)

Pearson correlation coefficients (r) with 95% confidence intervals (Fisher’s z-transformation) for the correlations shown in the metabolomics heatmap (Figure 3). n reflects the actual number of paired observations with non-missing data for that specific pair. NA indicates a correlation could not be calculated. Where a correlation coefficient is reported (r), but the confidence interval is NA, Fisher’s z-transformation is undefined at n ≤ 3 and a CI could not be estimated. Given the small subgroup sizes (n = 5–12 Pre-Frail, n = 3–6 Frail), these estimates carry substantial uncertainty and should be interpreted descriptively rather than as evidence of a biological transition between frailty states.

**Table A4:** 95% Confidence Intervals for Correlations Between Protein Abundance and Physical Function/Anthropometric Measures, by Frailty Status.

Anthropometric Measure	Analyte	n (Pre-Frail)	r (95% CI) Pre-Frail	n (Frail)	r (95% CI) Frail
BRS	MMP-19	12	0.03 (−0.55 to 0.59)	5	0.03 (−0.88 to 0.89)
BRS	COEA1	11	0.24 (−0.42 to 0.73)	5	0.03 (−0.87 to 0.89)
BRS	GSLG1	12	0.51 (−0.10 to 0.84)	5	−0.90 (−0.99 to −0.10)
BRS	S38AA	12	0.03 (−0.55 to 0.59)	6	−0.18 (−0.87 to 0.74)
BRS	FCRL5	12	0.13 (−0.48 to 0.66)	5	−0.30 (−0.94 to 0.79)
BRS	ATS1	11	NA	6	0.09 (−0.78 to 0.84)
BRS	PTX3	12	−0.23 (−0.71 to 0.39)	6	0.70 (−0.25 to 0.96)
BRS	4F2	11	0.11 (−0.52 to 0.67)	5	−0.47 (−0.96 to 0.71)
ADL	MMP-19	12	NA	6	0.27 (−0.70 to 0.89)
ADL	COEA1	12	NA	6	0.40 (−0.61 to 0.91)
ADL	GSLG1	12	−0.25 (−0.72 to 0.38)	5	0.06 (−0.87 to 0.89)
ADL	S38AA	9	0.39 (−0.37 to 0.84)	5	0.82 (−0.23 to 0.99)
ADL	FCRL5	12	NA	6	0.13 (−0.76 to 0.85)
ADL	ATS1	12	−0.44 (−0.81 to 0.18)	6	−0.39 (−0.91 to 0.62)
ADL	PTX3	12	−0.08 (−0.62 to 0.52)	6	0.10 (−0.77 to 0.84)
ADL	4F2	12	−0.19 (−0.69 to 0.43)	5	−0.01 (−0.88 to 0.88)
REAPS	MMP-19	12	0.13 (−0.48 to 0.65)	6	−0.11 (−0.85 to 0.77)
REAPS	COEA1	12	0.46 (−0.15 to 0.82)	6	−0.59 (−0.95 to 0.42)
REAPS	GSLG1	12	NA	6	−0.72 (−0.97 to 0.22)
REAPS	S38AA	12	−0.14 (−0.66 to 0.47)	4	−0.78 (−1.00 to 0.73)
REAPS	FCRL5	7	0.80 (0.11 to 0.97)	5	−0.14 (−0.91 to 0.85)
REAPS	ATS1	12	−0.15 (−0.67 to 0.47)	6	0.82 (0.02 to 0.98)
REAPS	PTX3	12	0.02 (−0.56 to 0.59)	4	−0.15 (−0.97 to 0.95)
REAPS	4F2	12	0.25 (−0.38 to 0.72)	5	0.14 (−0.85 to 0.91)
Weight	MMP-19	11	−0.51 (−0.85 to 0.13)	4	−0.49 (−0.99 to 0.89)
Weight	COEA1	7	−0.13 (−0.80 to 0.69)	3	1.00 (NA)
Weight	GSLG1	12	0.07 (−0.52 to 0.62)	5	0.13 (−0.85 to 0.91)
Weight	S38AA	11	0.55 (−0.07 to 0.86)	5	0.41 (−0.74 to 0.95)
Weight	FCRL5	12	−0.20 (−0.69 to 0.42)	5	−0.38 (−0.95 to 0.75)
Weight	ATS1	7	0.36 (−0.54 to 0.88)	3	−0.56 (NA)
Weight	PTX3	11	NA	5	0.60 (−0.60 to 0.97)
Weight	4F2	12	0.16 (−0.45 to 0.67)	6	−0.35 (−0.91 to 0.64)
Gait	MMP-19	10	0.38 (−0.32 to 0.82)	3	0.24 (NA)
Gait	COEA1	12	0.63 (0.09 to 0.88)	5	0.85 (−0.12 to 0.99)
Gait	GSLG1	8	−0.82 (−0.97 to −0.28)	4	0.10 (−0.95 to 0.97)
Gait	S38AA	10	0.51 (−0.18 to 0.86)	4	−0.62 (−0.99 to 0.85)
Gait	FCRL5	12	−0.13 (−0.66 to 0.48)	5	0.60 (−0.60 to 0.97)
Gait	ATS1	12	−0.11 (−0.65 to 0.49)	5	−0.82 (−0.99 to 0.23)
Gait	PTX3	10	NA	4	−0.50 (−0.99 to 0.89)
Gait	4F2	12	0.14 (−0.47 to 0.66)	6	0.05 (−0.79 to 0.83)

Pearson correlation coefficients (r) with 95% confidence intervals (Fisher’s z-transformation) for the correlations shown in the proteomics heatmap (Figure 4). n reflects the actual number of paired observations with non-missing data for that specific pair. NA indicates a correlation could not be calculated. Where a correlation coefficient is reported (r), but the confidence interval is NA, Fisher’s z-transformation is undefined at n ≤ 3 and a CI could not be estimated. Given the small subgroup sizes (n = 5–12 Pre-Frail, n = 3–6 Frail), these estimates carry substantial uncertainty and should be interpreted descriptively rather than as evidence of a biological transition between frailty states.
